# Antimicrobial and In Vitro Cytotoxic Efficacy of Biogenic Silver Nanoparticles (Ag-NPs) Fabricated by Callus Extract of *Solanum incanum* L.

**DOI:** 10.3390/biom11030341

**Published:** 2021-02-24

**Authors:** Islam Lashin, Amr Fouda, Adil A. Gobouri, Ehab Azab, Zuhair M. Mohammedsaleh, Rabab R. Makharita

**Affiliations:** 1 Botany and Microbiology Department, Faculty of Science, Al-Azhar University, Nasr City, Cairo 11884, Egypt; islam79@azhar.edu.eg; 2 Department of Biology, Faculty of Science and Arts, Al-Mandaq Al-Baha University, Al-Baha 1988, Saudi Arabia; 3 Department of Chemistry, College of Science, Taif University, P.O. Box 11099, Taif 21944, Saudi Arabia; a.gobouri@tu.edu.sa; 4 Department of Biotechnology, College of Science, Taif University, P.O. Box 11099, Taif 21944, Saudi Arabia; e.azab@tu.edu.sa; 5 Department of Medical Laboratory Technology, Faculty of Applied Medical Sciences, University of Tabuk, Tabuk 71491, Saudi Arabia; zsaleh@ut.edu.sa; 6 Biology Department, Faculty of Science and Arts, Khulais, University of Jeddah, Jeddah 21959, Saudi Arabia; rabab_makharita@science.suez.edu.eg; 7 Botany and Microbiology Department, Faculty of Science, Suez Canal University, Ismailia 41522, Egypt

**Keywords:** *Solanum incanum* L., silver nanoparticles, callus aqueous extract, antimicrobial, phytopathogenic fungi, in vitro cytotoxicity

## Abstract

The in vitro callus induction of *Solanum incanum* L. was executed on MS medium supplemented with different concentrations of auxin and cytokinin utilizing petioles and explants of leaves. The highest significant fresh weights from petioles and leaf explants were 4.68 and 5.13 g/jar for the medium supplemented with1.0 mg L^−1^ BA and 1.0 mg L^−1^ 2,4-D. The callus extract of the leaves was used for the green synthesis of silver nanoparticles (Ag-NPs). Analytical methods used for Ag-NPs characterization were UV-vis spectroscopy, Fourier Transform Infrared spectroscopy (FT-IR), X-ray diffraction (XRD), and Transmission Electron Microscopy (TEM). Spherical, crystallographic Ag-NPs with sizes ranging from 15 to 60nm were successfully formed. The FT-IR spectra exhibited the role of the metabolites involved in callus extract in reducing and capping Ag-NPs. The biological activities of Ag-NPs were dose-dependent. The MIC value for *Staphylococcus aureus*, *Bacillus subtilis*, and *Escherichia coli* was 12.5 µg mL^−1^, while it was 6.25 µg mL^−1^ for *Klebsiella pneumoniae*, *Pseudomonas aeruginosa,* and *Candida albicans*. The highest inhibition of phytopathogenic fungi *Alternaria alternata*, *Fusarium oxysporum*, *Aspergillus niger*, and *Pythium ultimum* was 76.3 ± 3.7, 88.9 ± 4.1, 67.8 ± 2.1, and 76.4 ± 1.0%, respectively at 200 µg mL^−1^. Moreover, green synthesized Ag-NPs showed cytotoxic efficacy against cancerous cell lines HepG2, MCF-7 and normal Vero cell line with IC_50_ values of 21.76 ± 0.56, 50.19 ± 1.71, and 129.9 ± 0.94 µg mL^−1^, respectively.

## 1. Introduction

Nanotechnology is a multidisciplinary science dealing with bioengineering, biology, physics, and chemistry. Nanoparticles (NPs) are substances of nano-size in the range of 1.0 to 100 nm [1]. Because of the unique properties of newly formed substances at the nanoscales, such as their small size, thermal conductivity, large surface area-to-volume ratio, and chemical steadiness, NPs can be integrated into different biomedical and biotechnological applications. Recently, NPs have been applied to medicine, food, animal feed, cosmetic substances, pharmaceuticals, the agricultural sectors, electrical fields, heritage preservation against harmful microbes, the textile industry, and optics [2,3,4,5]. Recently, NPs have been utilized for the adsorption of heavy metals, removal of textile dyes, and degradation of other environmental pollutants [6,7,8]. Metal and metal oxides NPs are synthesized via various chemicals and physical methods such as radiation, laser ablation, microwave, chemical reduction, sol-gel, sonochemical, and coprecipitation methods [9,10,11]. The major disadvantages of these methods include high costs, the use of hazardous chemicals, the demand for rigorous environmental conditions such as suitable temperature and pH, scalability difficulties, and the creation of toxic by-products [12]. Therefore, it is necessary to develop a green approach that is considers cost-effective, rapid, eco-friendly, scalable, and avoids the use of harsh conditions or toxic substances. Green synthesis, or biosynthesis, refers to the fabrication of NPs using biological systems such as microorganisms (bacteria, fungi, yeast, algae, actinomycetes, and viruses) and plants by harnessing their metabolites to reduce, cap, and stabilize NPs [13,14,15].

Silver nanoparticles (Ag-NPs) have received more attention in recent decades because of their safe applications in different biomedical and biotechnological sectors [16]. Recently, Ag-NPs have been employed as antimicrobial agents, for wound healing, for anti-viral, and anti-inflammatory purposes, for the control of phytopathogenic microbes, and as anti-cancer cells and, optical receptors. They have also been used as catalysts for chemical reactions, biosensors, as antioxidants, and for DNA delivery [17,18]. These diverse activities could be attributed to the unique new properties of Ag-NPs such as their stability, catalytic activity, biocompatibility, high conductivity, and large surface area-to-volume ratio [19]. The green synthesis of Ag-NPs can be achieved by harnessing the metabolites of bacteria, fungi, yeast, algae, actinomycetes, and plants as reducing and capping agents instead of the hazardous materials used in chemical and physical synthesis. Plant-mediated green synthesis of Ag-NPs has gained more attention because of its low-cost, simplicity, scalability, eco-friendliness, and the diverse of metabolites secreted by plants [20]. Blood can transport different foreign substances to tissues, cells, and other organs. Additionally, it can receive nanoparticles through inhalations or injection via mucosa or the skin and, hence, translocate them into the bloodstream. Moreover, various biomedical applications require an injection of NPs into the body and, hence, it directly contacts the bloodstream [21]. Therefore, the nanotoxicity on blood components should be investigated.

The flora of the Kingdom of Saudi Arabia (KSA) possesses approximately 1200 out of 2250 flowering plants that have medicinal importance [22]. *Solanum incanum* L. (family: Solanaceae) is one of the nightshade medicinal plants found in KSA and is endemic to sub-Saharan Africa, the Middle East, and India [23]. The *S. incanum* L. plant has been identified as a good source of different phytochemical compounds that can be used against pathogens, predators, and for the treatment of various human and animal diseases [24]. Based on its analgesic properties, this plant has been used to treat sore throat, stomach-ache, headache, angina, painful menstruation, colic, liver pain, pneumonia, rheumatism, and pleurisy [23]. Tissue culture refers to the in vitro cultivation of cells, organs, tissues, or the whole plant under aseptic and controlled environmental and nutritional conditions [25]. Recently, plant tissue culture techniques have been utilized as a significant tool for large-scale plant propagation, controlled plant disease, and improvement of the production of plant secondary metabolites. A small part of the tissue (explant) is used to propagate plants in a continuous process [26]. The use of the plant tissue culture technique avoids the exposure of the plant to biotic and abiotic stresses that affect the plant’s secondary metabolite production [27].

Therefore, the main objective of the current study was to assess the efficacy of the callus extract of the medicinal plant *S. incanum* L. to fabricate Ag-NPs. To attain this goal, the petioles and leaves of *S. incanum* L. were used as explant to propagate callus and select the high yield one for the green synthesis of Ag-NPs. Color change, UV-vis spectroscopy, Fourier Transform Infrared spectroscopy (FT-IR), X-ray Diffraction (XRD), and Transmission Electron Microscopy (TEM) were used for the physicochemical characterization of Ag-NPs. Moreover, the biological activities of the callus-mediated green synthesis of Ag-NPs including antimicrobial activity, antifungal activity against phytopathogenic fungi, and in vitro cytotoxicity against cancerous cells were investigated.

## 2. Materials and Methods

### 2.1. Plant Materials

**Figure 1:**
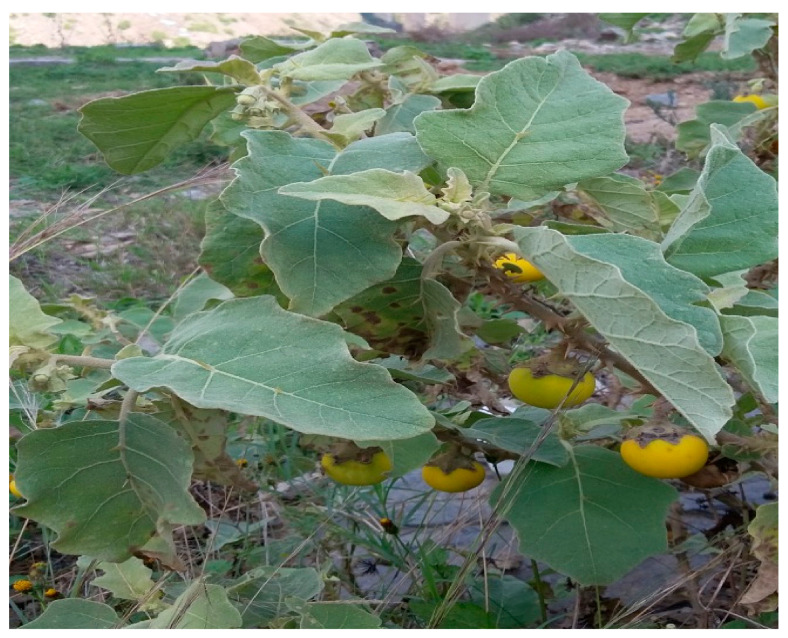
Field picture of *Solanum incanum* L. collects from Wadi Al Khilb, Al-Baha area, KSA.

*Solanum incanum* L. plants were collected from Wadi Al Khilb, Albaha, KSA (Figure 1). The primary identification of the collected plant was achieved in the field, while taxonomic identification was carried out by the herbarium academic staff of Albaha University, KSA. These plants were used as a source of plant materials (leaves and petioles). The explants were immersed in ethanol (70 %) for one min. followed by surface disinfection by via sodium hypochlorite (5.25 %) at different times and concentrations.

### 2.2. Callus Induction

The surface-sterilized leaves and petioles of *Solanum incanum* L. were cut into segments, each one approximately 1 cm^2^, and used as explants. The explants were cultured on Murashige and Skoog 1962 (MS) basal medium supplemented with sucrose (25.0 g L^−1^), Agar (7.0 g L^−1^), and growth regulators which Benzylaminopurine (BAP) (0.0, 0.5, 1.0, and 1.5 mg L^−1^) and 2,4-Dichlorophenoxyacetic acid (2,4-D) (0.0, 1.0, 1.5 and 2.0 mg L^−1^), pH was adjusted at 5.7 ± 1. All cultures were examined after 5 weeks of incubation at 26 ± 2 °C under light/dark conditions for 16/8 h, respectively with a light intensity of 1500 lux provided by white, fluorescent tubes. The callus induction frequency (%) is calculated using the following equation:

(1) $$Callus\;Induction\;Frequency\left(\%\right)=\frac{number\;of\;callus}{number\;of\;explants}\times 100$$

Fresh and dry weights of callus (g) were measured after five weeks of cultures and moisture content of callus cultures was estimated using the following equation:

(2) $$Moisture\;Content(\%)=\frac{fresh\;weight-dry\;weight}{fresh\;weight}\times 100$$

### 2.3. Preparation of Callus Aqueous Extract of S. incanum L.

Fresh biomass of callus was collected, washed thrice with sterile distilled water (dis. H_2_O), and dried in the hot air oven at 40 °C for 24 h. About five grams of fine powder was added to a 250 mL conical flask containing 100 mL of sterile dis. H_2_O and boiled for 30 min at 80 °C. The previous mixture could cool and then filtered using a Whatman No. 1 filter paper. The filtrate (callus aqueous extract) was collected and utilized after that to form Ag-NPs. The phytochemical constituents of callus extract were conducted to detect the presence of alkaloids, flavonoids, glucosides, and terpenoids according to Sbhatu and Abraha [28] and Sofowara [29]. Briefly, 1.0 mL of callus extract was mixed with 5.0 mL of HCl (1%) and stirred in a steam bath. The mixture was cooled, filtered, and a few drops of Wanger’s reagent (2.0 g of iodine mixed with 6.0 g of potassium iodide in 100 mL dis. H_2_O) were added to 1.0 mL of previous filtrate to detect the alkaloids via formation of yellow or brown precipitate. Meanwhile the presence of flavonoids was detected by adding 5 mL of diluted HCl to 0.5 mL of callus extract and Mg powder, boiled for 5.0 minutes to form a brown or reddish pink color. In addition, glycosides were detected by mixing callus extract with aqueous NaOH solution to form a yellow color. Terpenoids were detected by mixing 5 mL of callus extract to 2 mL of chloroform, followed by adding 3 mL of Conc. H_2_SO_4_ to form a reddish color.

### 2.4. Aqueous Callus Extract Mediated Green Synthesis of Ag-NPs

The aqueous callus extract was used for Ag-NPs synthesis as follows: about 10 mL of callus extract was mixed with 90 mL of 1.0 mM AgNO_3_ (grade, Sigma Aldrich, Munich, Germany) and incubated in dark condition at 35 °C for overnight [13]. The color change of the reaction mixture was monitored. The Ag-NPs were collected by centrifugation at 10,000 rpm for 15 min. The pellet was oven-dried at 80 °C for 48 h and used for characterization.

### 2.5. Analytical Methods for Characterization of Ag-NPs

#### 2.5.1. UV-Visible Spectroscopy

The formation of Ag-NPs was confirmed by the color change of callus extract after mixed with NPs precursor (AgNO_3_) was investigated at the first visually followed by a measure of the absorbance at a wavelength range between 300 and 600 nm to distinguish the maximum surface plasmon resonance (SPR). To achieve this goal, the UV-Vis spectra were measured using JENWAY 6305 spectrophotometer.

#### 2.5.2. Fourier Transform Infrared Spectroscopy (FT-IR)

The functional groups present in callus aqueous extract that have important roles in the biofabrication of Ag-NPs were analyzed using FT-IR. Agilent system Cary 630 FT-IR model was used to achieve this goal by measure the wavenumber in the range 400 to 4000 cm^−1^ [30].

#### 2.5.3. X-ray Diffraction (XRD)

Phase formation, purity, and crystalline nature of synthesized Ag-NPs were determined using an X-ray diffraction spectrometer (Philips, Eindhoven, The Netherlands). The process was achieved at a 2θ value between 20° and 80°. Additionally, X-ray source, voltage, and current were Ni-filtered Cu/Ka radiation, 40 KV, and 30 mA, respectively. The particle Ag-NPs size can be calculated as a result of XRD analysis through the Scherrer equation as follows:

(3) Average NPs size(D)=0.9λ/β Cosθ

where 0.9 is Scherrer constant; λ is X-ray wavelength; β is the half-maximum intensity, and θ is the Bragg’s angle.

#### 2.5.4. Transmission Electron Microscopy (TEM)

The TEM instrument (JEOL, JEM-1230, Tokyo, Japan) with voltage acceleration 120 KV was used to detect the size and shape of biosynthesized Ag-NPs. A drop of colloidal Ag-NPs solution was loaded on a carbon-coated copper TEM grid. The loaded gride is touched with blotting gride to remove the excess of NPs solution before placed onto the gride TEM box [31].

#### 2.5.5. Energy Dispersive Spectroscopic Analysis (SEM-EDX)

The elemental compositions of Ag-NPs synthesized by callus extract was detected by using SEM (type: JEOL, JSM-6360LA, Japan) connected with energy dispersive spectroscopy (EDX) instrument.

### 2.6. Biological Activities of Green Synthesized Ag-NPs

#### 2.6.1. Antimicrobial Activities

The agar well diffusion method used to evaluate the antimicrobial activities of green synthesized Ag-NPs against *Staphylococcus aureus* ATCC6538, *Bacillus subtilis* ATCC6633 as Gram-positive bacteria, *Escherichia coli* ATCC8739, *Klebsiella pneumoniae* ATCC 700603, *Pseudomonas aeruginosa* ATCC9022 as Gram-negative bacteria, and *Candida albicans* ATCC10231 as unicellular fungi. Briefly, each purified species was streaked over Muller Hinton agar media using a sterilized cotton swab. After that, wells (0.7 mm diameter) were filled with100 µL of stock Ag-NPs solution (200 ppm). To calculate minimum inhibitory concentration (MIC), different concertation (100.0, 50.0, 25.0, 12.5, 6.25, and 3.125 ppm) were prepared. The loaded plates were kept in the refrigerator for about one hour before incubated at 37 °C overnight. The results were measured as a diameter of inhibition zone (mm) that formed around each well [32]. The experiment was performed in triplicate.

#### 2.6.2. Antifungal Activities against Phytopathogen Fungi

Plant pathogenic fungi represented by *Fusarium oxysporum, Alternaria alternata, Aspergillus niger, and Pythium ultimum* were isolated and identified by helping Dr. Samir A. M. Mahgoub (Plant Pathology Department, Faculty of Agriculture, Zagazig University). The efficacy of green synthesized Ag-NPs to inhibit the growth of collected phytopathogenic fungi was investigated as follows: potato dextrose agar (PDA) media mixed with 100 µL of different Ag-NPs concentrations (200, 150, 100, 50, and 25 µg mL^−1^) before solidification. After that, the fungal agar plug (0.7 cm in diameter) of four growing old days was transferred to previous PDA media. The plates were incubated at 25 °C ± 2 °C for five days. The results were recorded as inhibition percentages (%) for fungal radial growth of control (growth without adding Ag-NPs) and fungal radial growth of treatment (growth after adding Ag-NPs) based on the following equation [33]

(4) $$Inhibition\;percentages(\%)=\frac{Control\;radial\;growth-Treatment\;radial\;growth}{Control\;radial\;growth}\times 100$$

#### 2.6.3. In Vitro Cytotoxic Efficacy of Ag-NPs against Cancerous Cells

Two cancerous cells, namely, Hep-G2 (human liver cancerous cell) and MCF-7 (breast cancer cell), and one normal cell line, Vero (kidney of African green monkey) were obtained from ATCC (American type culture collection) and used to assess the in vitro cytotoxic efficacy of green synthesized Ag-NPs by MTT [3-(4,5-dimethylthiazol-2-yl)-2,5-diphenyl tetrazolium bromide] assay method. Briefly, the two cancerous cells were cultivated in 96-well culture plate (at concentration 1× 10^5^ cell/mL) and treated by biosynthesized Ag-NPs at concentrations 200, 150, 100, 75, 50, 25, 12.5, 6.25, and 3.125 µg/mL. The treated plates were incubated at 37 °C for 48 h. After that, MTT (5 mg mL^−1^ in phosphate buffer solution) was mixed with each well and incubated for 1–5 h/5% CO_2_ at 37 °C. At the end of the incubation period, the purple formazan crystal was formed, which were dissolved after that by adding 10% dimethyl sulfoxide (DMSO) under an agitating state for 30 min in dark condition. Finally, the intensity of the formed color was measured at 560 nm by an ELIZA plate reader. As a part of the experiment, a plate inoculated with cancerous cells without Ag-NPs treatment was running as control. The cell viability percentages (%) was calculated according to the following formula [34]:

(5) $$Cell\;viability\;percentages(\%)=\frac{Treated\;absorbance}{Control\;absorbance}\times 100$$

### 2.7. Statistical Analysis

All results presented in this study are the means of three independent replicates. Data were subjected to analysis of variance (ANOVA) by a statistical package SPSS v17. The mean difference comparison between the treatments was analyzed by the Tukey HSD test at *p* < 0.05.

## 3. Results and Discussion

### 3.1. Effect of Plant Growth Regulators on Callus Initiation

Recently, biotechnology has offered attractive opportunities for the production of plant-based in vitro systems (e.g., callus cultures). Plant cell cultures represent interesting sources for the easy and scalable production of secondary metabolites. Callus culture technquies are used to increase the concentrations of secondary metabolites that are activated by elicitors and released as defense responses [35]. Tissue culture techniques provide continuous, reliable, and renewable sources of valuable plant pharmaceuticals and might be used for the large-scale culture of the plant cells from which these secondary metabolites can be extracted. The major advantages of cell culture systems, as compared with conventional cultivation, include the fact that the plant compounds of choice can be generated independently of external factors (e.g., soil composition or climate); also, callus culture systems reduce the contamination sources and biotic and abiotic stresses that may affect plant growth in a normal environment [35].

Phytochemical screening tests of the leaf and petiole crude extracts of *S. incanum* gave positive results for alkaloids, saponins, flavonoids, glycosides, terpenoids, and steroids, so we used the leaf and petiole explant in this study [28].

**Figure 2:**
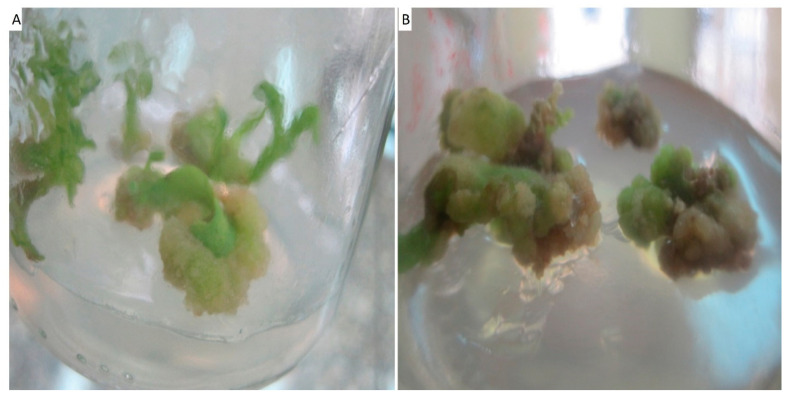
Callus induction from leaves (**A**) and petioles (**B**) of *Solanum incanum* L. after five weeks of culture on MS medium supplemented with 2.4-D (1.0 mg L^−1^) and BA (1.0 mg L^−1^).

In this study, the leaf and petiole explants when cultured on MS basal medium supplemented with different concentrations of growth regulators showed the induction of callus. Data represented in Table 1 and Figure 2 show the callus induction frequency from leaves and petioles of *Solanum incanum* L. after 5 weeks of growth. Callus was induced from all explants (leaves and petioles) in all treatments except on the control medium (without growth regulators). The highest percentages of callus formation were 90% and 86.6% for leaves and petioles, respectively, cultured on MS medium with 1.0 mg L^−1^ of BA and 1.0 mg L^−1^ of 2,4-D. These findings are in agreement with Tang et al. [36], who recorded that the highest percentages of callus formation were obtained from the leaves and petioles of *Lilium leucanthum* cultured on MS medium containing BA (1.0 mg L^−1^) and l 2,4-D (1.0 mg L^−1^).

Data in Table 2 show the effect of MS medium supplemented with different concentrations of BA and 2,4-D on the fresh weight, dry weight (g/jar), and moisture content (%) of calli produced from leaves and petioles of *Solanum incanum* L. after five weeks of culture. Data analysis showed that the measured parameters significantly decreased by increasing growth regulators. The lowest values of fresh weight, dry weight, and moisture content for explants originating from petioles were 0.9 g/jar, 0.08 g/jar, and 91.11%, respectively, because of the addition of 1.5 mg L^−1^ BA and 2.0 mg L^−1^ 2,4-D to the MS medium. Moreover, the highest dry weight values of calli (0.37 g/Jar) were recorded after five weeks of cultivation with a leaf on MS medium supplemented with1.0 mg/L BA and 1.0 mg/L 2,4-D. On the other hand, the highest significant fresh weights from explants petioles and leaves were 4.68 and 5.13 g, recorded for the medium supplemented with1.0 mg L^−1^ BA and 1.0 mg L^−1^ 2,4-D, respectively. Supporting evidence for these results was recorded on *Tanacetum parthenium*; after using 2,4-D, NAA, or BAP at 0.0, 0.5 or 1.0 mg L^−1^ individually or in combination form, applying the mixture of 1.0 mg L^−1^ NAA plus 1.0 mg L^−1^ BAP resulted in the heaviest fresh and dry callus weight [37].

### 3.2. Preparation of Aqueous Callus Extract and Phytochemical Screening

In the present study, five-week-old, compact, hard callus from the leaf explants of *S. incanum* L. was used to obtain the callus extract (based on high yield) as mentioned in Section 2. The phytochemical screening of this callus extract was investigated for the presence of different constituents. Data showed that the callus aqueous extarct of *S. incanum* L. gave positive results for alkaloids, flavonoids, glucosides, and terpenoids in callus aqueous extract of *S. incanum* L. These constituents play a vital role in reducing, capping, and stabilizing of Ag-NPs. Consistent with the present data, Sbhatu and Abraha [28] showed that the extract of leaf, fruit, and stem of *S. incanum* were positive for saponins, glycosides, alkaloids, terpenoids, flavonoids, and steroids. Marslin et al. [1], reported that plant aqueous extract utilized for green synthesis of NPs contains a wide range of metabolites such as protein, flavonoids, terpenoids, alcoholic compounds, polyphenolic compounds, various organic acids, polysaccharides, and others. These metabolites have important roles in reducing metal or metal oxide ions to NPs as well as supporting stability to these products [38]. The efficacy of terpenoid present in leave extracts of *Andrographis paniculate* utilized for biosynthesis of zinc oxide NPs (ZnO-NPs) has been verified and confirmed as by C=O functional groups as detected by FT-IR analysis [39].

### 3.3. Callus Aqueous Extract Mediated Green Synthesis of Ag-Nps

#### 3.3.1. Color Change and UV-Vis Spectroscopy

In the current study, the collected leaf callus extracts were utilized as a biocatalyst for the green synthesis of Ag-NPs. The first indicator of the successful fabrication of Ag-NPs using callus extract was a color change from pale yellow to yellowish-brown. This color change could be attributed to the reduction of Ag^+^ to metallic nano-silver (Ag^o^) via the activities of the metabolites involved in the callus extract. Recently, the color of aqueous fruit extract of *Solanum incanum* L. was changed to brown after mixed with AgNO_3_ because of the formation of Ag-NPs [40]. Additionally, the color of *Hyptis suaveolens* callus extract changed from pale yellow to deep brown due to mixing with AgNO_3_ and the formation of Ag-NPs [13]. The formation process involved an electron donation from the metabolites involved in callus extract, which reduced the metal ions to nanoparticles. The as-formed NPs were synthesized as a consequence of the high surface energy, and by preventing aggregations, were converted to the same conformations when reaching the low surface energy. Therefore, large amounts of reducing and stabilizing substances in the filtrates prevented the aggregations of NPs and enhanced the production of smaller NPs sizes [1]. Moreover, proteins present in the aqueous extract imprisoned the metals on the surface and changed them to nuclei, which then aggregated to form specific nanoparticles [38].

**Figure 3:**
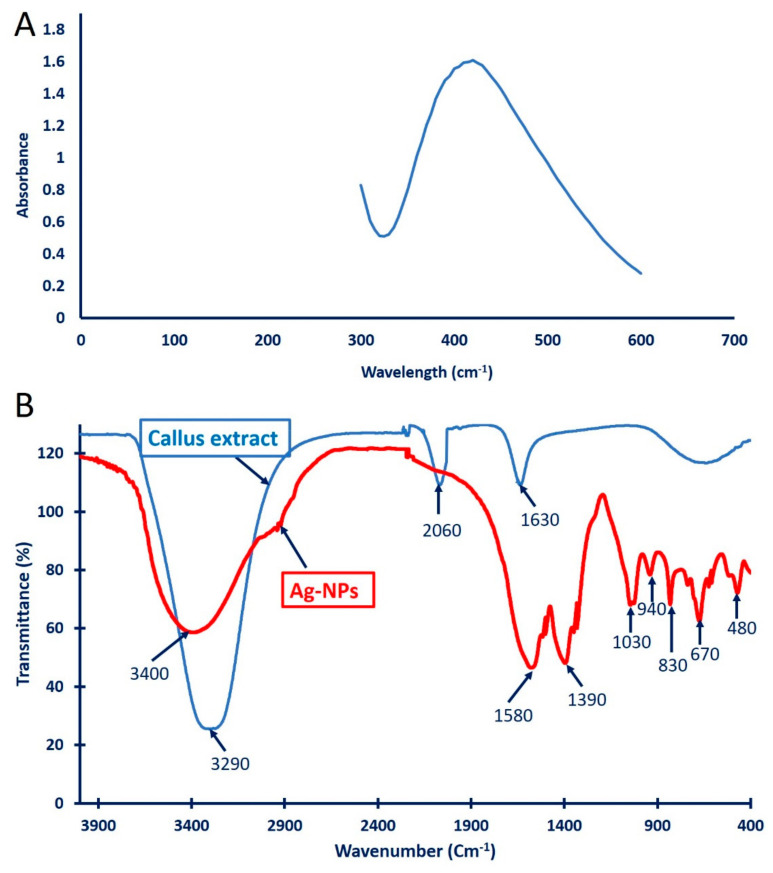
Characterization of green synthesized Ag-NPs using callus aqueous extract of *S. incanum* L. (**A**) UV-vis spectroscopy of biosynthesized Ag-NPs; (**B**) FT-IR spectra of callus aqueous extract and green synthesized Ag-NPs.

The successful fabrication of Ag-NPs was confirmed when detecting the surface plasmon resonance (SPR) via UV-vis spectroscopy at wavelengths of 300 to 600 nm. As shown in Figure 3A, the SPR of the callus mediated biosynthesis of Ag-NPs was observed at 440 nm. Consistent with our study, Mude et al. [41] reported the efficacy of *Carica papaya* callus extracts to fabricate Ag-NPs at an SPR value of 440 nm. The obtained data are incompatible with those obtained by Aref and Salem [42], who reported that the SPR for Ag-NPs synthesized with callus extract of *Cinnamonum camphora* was detected at 420 nm, while the SPR for Ag-NPs synthesized with callus extract of *Hyptis suaveolens* was observed at 447 nm [13]. Moreover, the SPR of Ag-NPs synthesized with the cold and hot water fruit extract of *Solanum incanum* was observed at 428.66 and 445.73 nm, respectively [40]. Different published studies reported that the SPR value of biosynthesized Ag-NPs was located between 400 and 450 nm and any shifting in this value may be attributed to the metabolites involved in the filtrates, which serve as reducing and stabilizing agents [43,44,45].

#### 3.3.2. Fourier Transform Infrared Spectroscopy (FT-IR)

The functional groups for different biomolecules responsible for the reduction of silver (Ag^+^) ions and capping and stabilizing reduced Ag-NPs were defined using FT-IR analysis (Figure 3B). The callus aqueous extracts showed three intense peaks at 3290, 2060, and 1630 cm^−1^. The strong and broad peak observed at 3290 cm^−1^ could be attributed to C-H stretching for alkyne and O-H stretching for carboxylic acid [46]. The peak at 2060 cm^−1^ signified the CO stretching for the carboxylic and unsaturated ester compounds, while the peak at 1630 cm^−1^ corresponded to C=O, C=N, and C=C for carboxylic, carbonyl, and I and II amide peptide linkage [47,48]. On the other hand, the FT-IR spectra of green synthesized Ag-NPs showed eight varied peaks (Figure 3B). The peak observed at 3400 cm^−1^ corresponded to the N-H stretching of aliphatic primary amines or the O-H stretching of alcohol [49]. The shifting peak at a wavenumber of 1580 cm^−1^ signified the bending vibration of the proteins (amide I), while the peak at 1390 cm^−1^ corresponded to the C-H bending of aldehyde [42]. The deformation of the O-H/C-O stretching of the phenolic/alcoholic groups was represented by a wavenumber of 1030 cm^−1^ [50]. Huang et al. [48] reported that the observed peak at 1032 cm^−1^ could be attributed to the absorption of C-O-C or C-O. The stretching peak appeared at 845 cm^−1^ may be related to a substitution on the aromatic ring, confirming the presence of proteins and phenolic compounds in callus extract, as mentioned previously [13]. The observed peak at 480 cm^−1^ indicates the efficacy of Ag-NPs binding with the OH group [51]. The presence of these vibrating bands confirmed the reduction and capping of Ag with the metabolites present in the callus extract.

#### 3.3.3. X-ray Diffraction (XRD)

**Figure 4:**
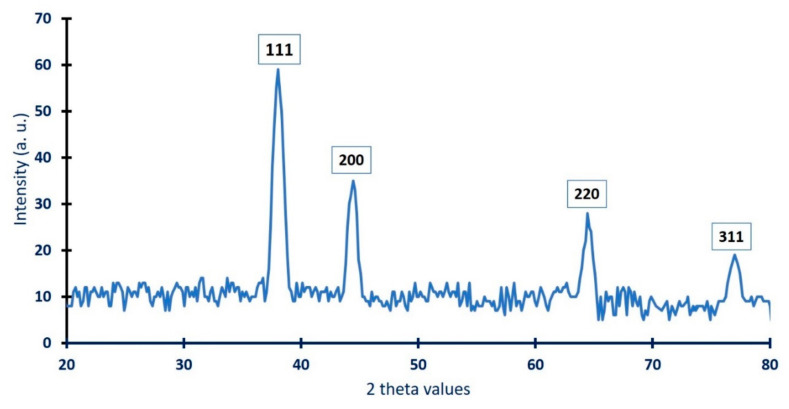
XRD pattern of Ag-NPs synthesized by callus aqueous extract of *S. incanum* L.

The crystallographic structure of Ag-NPs synthesized with the callus extract of *S. incanum* was investigated using XRD analysis. As shown in Figure 4, the biogenic Ag-NPs revealed four intense planes at 2*θ*° of 38.2°, 44.5°, 64.4°, and 77.02°, which corresponded to lattice planes (111), (200), (220), and (311), respectively. The obtained XRD data were compatible with JCPDS standard No. 04-0783, which confirmed that the biosynthesized Ag-NPs were crystallographic, face-centered cubic (FCC) structures [52,53]. Consistent with our study, Botcha, and Prattipati [13] reported the successful fabrication of crystalline Ag-NPs due to the reduction of Ag ions by metabolites present in the callus extract of *Hyptis suaveolens*, and characterized by face-centered cubic structures because of the presence of a diffraction peak at 2*θ*° of ≈38° (111). The broadening of the bases of the diffraction peaks indicates the successful fabrication of small Ag-NPs [54]. Moreover, the sharp diffraction peaks observed in the XRD spectra could be attributed to the stabilization of NPs because of the capping agents [13]. The presence of unassigned diffraction peaks in the XRD spectra may be related to the crystallization of biomolecules coating the surface of the NPs [55].

The sharp diffraction peak at lattice plane (111) confirmed the successful formation of nanosize particles. Their size (D) was calculated using the Scherrer equation. The data showed that the average Ag-NPs size synthesized via harnessing the metabolites of callus extract was 48 nm. The data are compatible with those obtained by Jemal et al. [56], who calculated using XRD analysis the size of Ag-NPs synthesized via leaf extract and callus extract of *Allophylus serratus* was 42 and 45 nm, respectively.

#### 3.3.4. Transmission Electron Microscopy (TEM)

**Figure 5:**
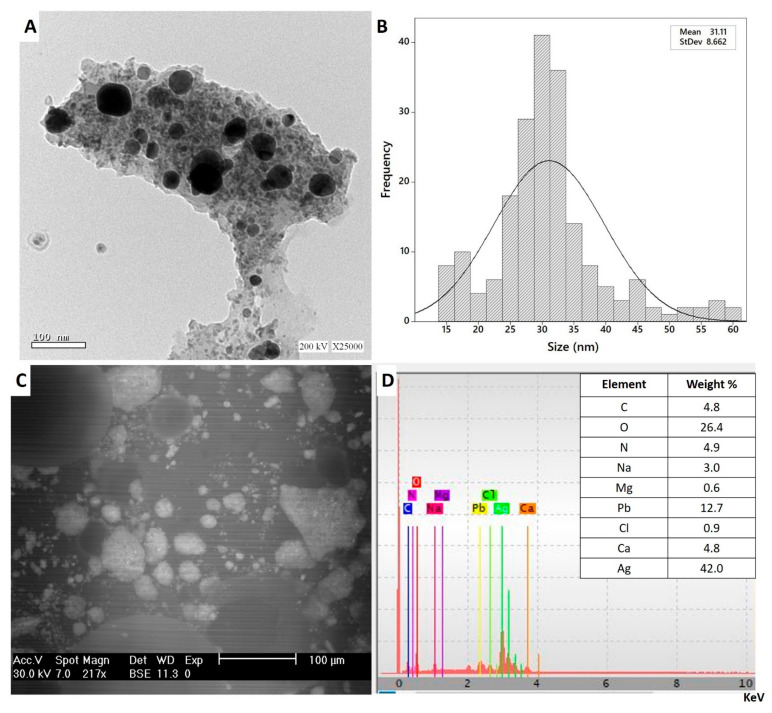
(**A**) Transmission Electron Microscopy (TEM) image; (**B**) particle size distribution; (**C**)Scanning Electron Microscopy (SEM); (**D**) EDX spectrum of Ag-NPs synthesized by callus extract of *S. incanum* L.

The biotechnological and biomedical activities of NPs are dependent on different parameters such as shape, size, and the distribution of NPs [57]. As the size decreased, the activities and biocompatibilities of NPs increased [58]. Therefore, the morphological characteristics of NPs should be examined. To attain this goal, a transmission electron microscopy (TEM) was used. The TEM image Figure 5A showed that the as-formed Ag-NPs were spherical, with sizes ranging from 15 to 60 nm with a mean ≈ of 31.1 nm (Figure 5B), which was smaller than those formed with plant extracts such as *Psoralea corylifolia* (100–110 nm), *Alternanthera dentate* (50–100 nm), and *Salvia spinosa* (19–125 nm) [59,60]. The obtained data are compatible with other published studies noting that the size of Ag-NPs synthesized using the callus extract of *Allophylus serratus* was 50 nm [56]. Additionally, the size of spherical Ag-NPs synthesized by harnessing metabolites of *Carica papaya* callus extract was in the range of 60 to 80 nm and suggested that the large as-formed size of the NPs could be attributed to capping proteins [41]. The TEM image confirms the efficacy of metabolites involved in callus extract to reduce and cap monodispersed spherical Ag-NPs without aggregations.

#### 3.3.5. Energy Dispersive Spectroscopic Analysis (SEM-EDX)

The qualitative as well as quantitative elemental compositions of Ag-NPs synthesized with callus extract were measured by EDX analysis. As shown, the metabolites involved in callus extract have the efficacy to fabricate spherical, monodispersed Ag-NPs (Figure 5C). The EDX spectra confirms the presence of Ag in the samples with weight percentages 42.0% (Figure 5D). Moreover, the presence of Ag-NPs peak at 3 KeV indicates that the silver occupied major components in the sample as mentioned previously [61]. The presence of optical absorption peak of Ag-NPs in EDX profile nearly to 3 KeV could be related to their SPR ([62]. The presence of N in EDX spectra could be related to the precursors (AgNO_3_). On the other hand, the presence of other elements such as O, C, Na, and Mg may be attributed to the scattering of callus metabolites that coating Ag-NPs such as proteins, carbohydrates, and amino acids by X-ray emissions [63].

### 3.4. Biological Activities of Green Synthesized Ag-NPs

#### 3.4.1. Antimicrobial Activities

Silver nanoparticles (Ag-NPs) are characterized by their efficacy to integrate into different biomedical and biotechnological applications because of their unique nanosize properties [45,64]. In this study, the antimicrobial activities of Ag-NPs synthesized by harnessing metabolites of *S. incanum* L. callus extract were examine against pathogenic Gram-positive (*Staphylococcus aureus* and *Bacillus subtilis*), Gram-negative bacteria (*Escherichia coli*, *Klebsiella pneumoniae*, and *Pseudomonas aeruginosa*), and unicellular fungi (*Candida albicans*) using agar well diffusion methods (Figure S1). The activities were assessed as a zone of inhibition (ZOI) formed around each well. Data analysis showed that the activities of Ag-NPs against bacterial and *Candida* sp. were dose-dependent. Consistent with this data, Eid et al. [34] reported that the activities of spherical Ag-NPs synthesized by endophytic *Streptomyces laurentii* against pathogenic bacterial species were dose-dependent.

**Figure 6:**
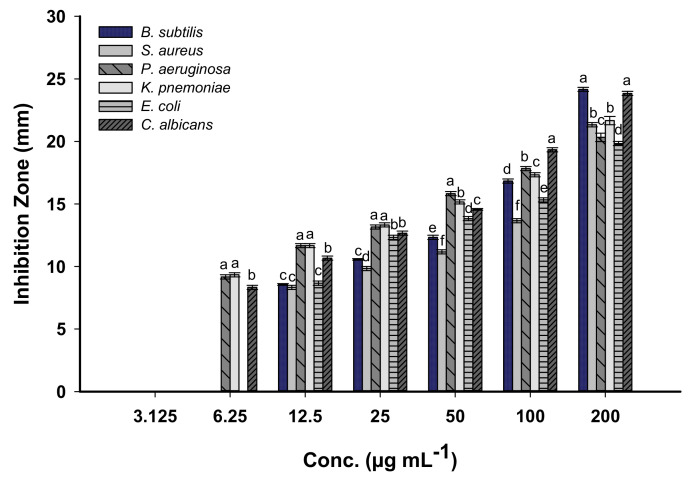
The antimicrobial activity of Ag-NPs synthesized by callus extract of *S. incanum* L. Data are statistically different at *p* ≤ 0.05, (*n* = 3); error bars are means ± SD (Standard deviation). For each treatment, bars with different letters indicate significantly different values at a significance level of *p* ≤ 0.05.

At high concentration of NPs (200 µg mL^−1^), the highest ZOIs were formed against *B. subtilis* and *C. albicans* with values of 24.2 ± 0.3 and 23.8 ± 0.3 mm, respectively. Meanwhile, the ZOIs that formed due to the same concentration of Ag-NPs against *S. aureus*, *P. aeruginosa*, *K. pneumoniae*, and *E. coli* were 21.3 ± 0.3, 20.3 ± 0.5, 21.7 ± 0.7, and 19.8 ± 0.3 mm, respectively. As the concentrations decreased, the antimicrobial activities also decreased. At lower Ag-NPs concentrations (100 µg mL^−1^), the values of ZOIs decreased to 16.8 ± 0.3, 13.7 ± 0.2, 17.8 ± 0.3, 17.3 ± 0.3, 15.3 ± 0.1, and 19.2 ± 0.2 mm for *B. subtilis*, *S. aureus*, *P. aeruginosa*, *K. pneumoniae*, *E. coli*, and *C. albicans*, respectively (Figure 6). Shkryl et al. [16] reported that the antimicrobial activity of Ag-NPs synthesized by callus extract of *Nicotiana tabacum* was enhanced by increasing the concentrations of NPs. Moreover, the inhibition zones that formed due to the treatment of multi-drug resistant pathogens *S. aureus* and *P. aeruginosa* with different concentrations (25, 50, 100, and 150 µL) of Ag-NPs synthesized via *Catharanthus roseus* callus extract were (4.0, 7.0, 16.0, and 23.0 mm) and (5.0, 9.0, 19.0, and 29.0 mm), respectively [65].

Data analysis showed that all tested pathogenic microbes are sensitive to low concentrations of Ag-NPs (12.5 µg mL^−1^) recording ZOI values of 8.6, 8.3, 11.7, 11.9, 8.7, and 10.7 mm for *B. subtilis*, *S. aureus*, *P. aeruginosa*, *K. pneumoniae*, *E. coli*, and *C. albicans*, respectively. The minimum inhibitory concentration (MIC) is defined as the lowest concentration that inhibits microbial growth. The MIC values should be detected for each bioactive compound. In the present study, the MIC value of biosynthesized Ag-NPs was 12.5 µg mL^−1^ for Gram-positive bacteria *B. subtilis*, *S. aureus*, and Gram-negative *E. coli*, recording ZOI values with an average of 8.5 mm. The MIC for Gram-negative bacteria *P. aeruginosa*, *K. pneumoniae*, and unicellular fungi *C. albicans* was 6.25 µg mL^−1^ with ZOI values of 9.1, 9.3, and 8.3 mm, respectively. The obtained data confirm the activities of Ag-NPs fabricated with metabolites of *S. incanum* L. against pathogenic prokaryote and eukaryote organisms at low concentrations. In our recent study, AgNO_3_ as positive control exhibit antimicrobial activity at high concnetrations. They showed ZOIs 11.3, 13.3, 10.3, 14.6, and 12.6 mm for *B. subtilis*, *S. aureus*, *E. coli*, *P. aeruginosa*, and *C. albicans*, respectively, for 2 mM concnetrations [66].

The prospective inhibitory effects of Ag-NPs could be attributed to the disruption of the cell wall and/or cell membrane, destroying the intracellular components because of entrance of NPs and enhancing oxidative stress [67,68]. In the present study, Gram-negative bacteria are more sensitive to the inhibitory effect of Ag-NPs than Gram-positive bacteria. This phenomenon may be attributed to the structure of the bacterial cell wall, which in Gram-negative bacteria contain lipopolysaccharides (negative charge) which are attracted to the positive charge of the NPs and hence disrupt the permeability functions of the cell membrane by reacting with sulfur and phosphorylated cell wall proteins [69]. As a result of the disrupted cell permeability, the NPs entering the bacterial cell and interact with the thiol groups of amino acids and thereby inactivate enzyme function. Additionally, Ag-NPs interact with nucleic acids and convert them from normal to a condensed state, thereby inhibiting DNA replications [70]. Moreover, Ag^+^ ions are liberated because of the entrance of Ag-NPs into the microbial cells, enhancing the reactive oxygen species (ROS) which destroy the cellular respiration system and ultimately lead to cell death [71].

#### 3.4.2. Antifungal against Phytopathogen Fungi

**Figure 7:**
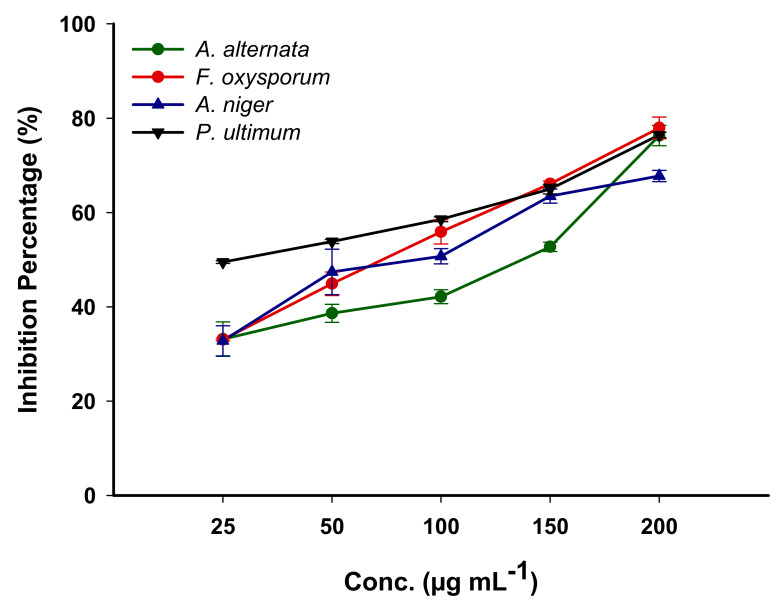
The activity of Ag-NPs synthesized using the callus extract of *S. incanum* L. against phytopathogenic fungi. Data are statistically different at *p* ≤ 0.05, (*n* = 3); error bars are means ± SD (standard deviation). The standard deviation is less than the size of the symbols if no error bars are seen.

Recently, the integration of NPs in agriculture sectors either to protect plants against pathogens or increase crop yields, has received more attention [67,72]. Fungi are eukaryotic microorganisms characterized by their efficacy to attack plant tissues and colonize them through various strategies [73]. The hypothesis that green synthesized Ag-NPs inhibit the growth of four pathogenic fungi isolated from an infected plant was investigated. To attain this hypothesis, different concentrations of Ag-NPs (200, 150, 100, 50, and 25 µg mL^−1^) were assessed against *Fusarium oxysporum*, *Alternaria alternata*, *Aspergillus niger*, and *Pythium ultimum* (Figure S2). An analysis of variance showed that the ability of Ag-NPs to inhibit the growth of pathogenic fungi can increase with increasing concentrations of NPs because of their biocompatibility and nontoxic nature [18]. At lower concentration (25 µg mL^−1^), the biosynthesized Ag-NPs decrease fungal growth with percentages of 33.1 ± 6.3, 33.0 ± 5.7, 32.8 ± 5.5, and 49.5 ± 0.5 % for *A. alternata*, *F. oxysporum*, *A. niger*, and *P. ultimum*, respectively. The highest level of fungal growth inhibition was achieved at Ag-NPs concentration 200 µg mL^−1^, which causes inhibition percentages of 76.3 ± 3.7, 88.9 ± 4.1, 67.8 ± 2.1, and 76.4 ± 1.0 % for *A. alternata*, *F. oxysporum*, *A. niger*, and *P. ultimum*, respectively (Figure 7).

Data analysis showed that fungal strain *P. ultimum* was highly sensitive to biosynthesized Ag-NPs. Recently, Ag-NPs fabricated via different endophytic *Streptomyces* species namely *S. capillispiralis* Ca-1, *S. zaomyceticus* Oc-5, and *S. pseudogriseolus* Acv-11, have the efficacy to inhibit the growth of phytopathogen *P. ultimum* with percentages of 52.5, 60.6, and 66.1%, respectively, at NPs concentrations of 2 mM [66]. Additionally, Ag-NPs synthesized via *Streptomyces* sp. exhibit high efficacy to inhibit the growth of phytopathogenic fungi *A. niger*, *A. flavus,* and *A. fumigatus* [74]. The obtained results regarding the potentiality of green synthesized Ag-NPs to inhibit the growth of phytopathogenic fungi are compatible with various published studies that prove the Ag-NPs possess more potent activities against pathogenic fungi when their concentration is increased [18,66,75,76]. The inhibitory mechanisms of Ag-NPs could be attributed to the liberation of fungal cell components as a result of the destruction of the fungal cell wall [77]. Moreover, the Ag-NPs can inhibit reproduction processes such as budding due to the dissipation of the electrical potential of the fungal membrane because of the formation of pits or pore-like structures [75].

#### 3.4.3. In Vitro Cytotoxicity

**Figure 8:**
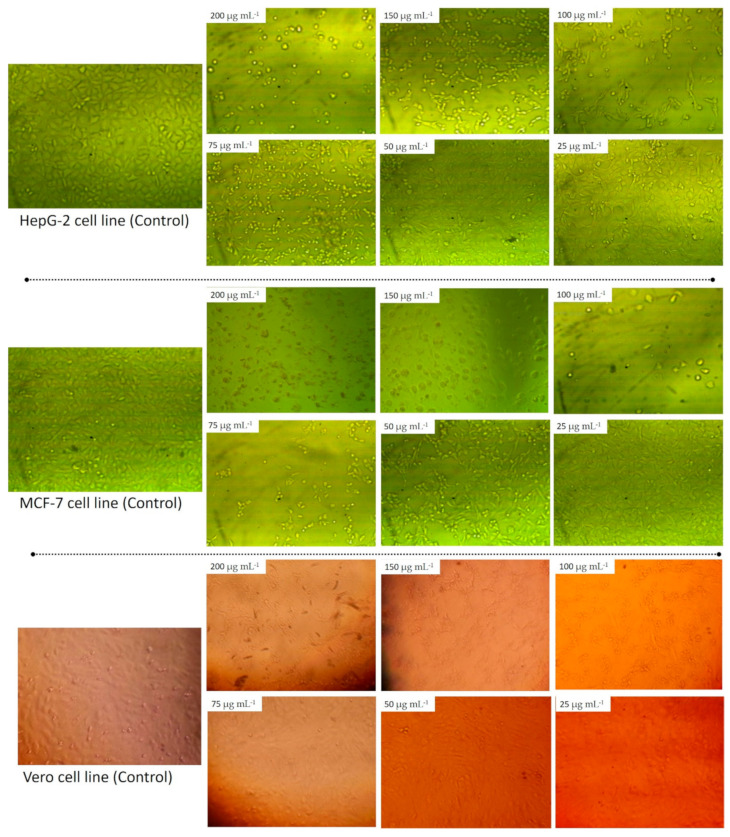
Morphological change of two cancerous cells, HepG2 and MCF-7 due to exposure to different concentrations of Ag-NPs synthesized by callus aqueous extract of *S. incanum* L.

**Figure 9:**
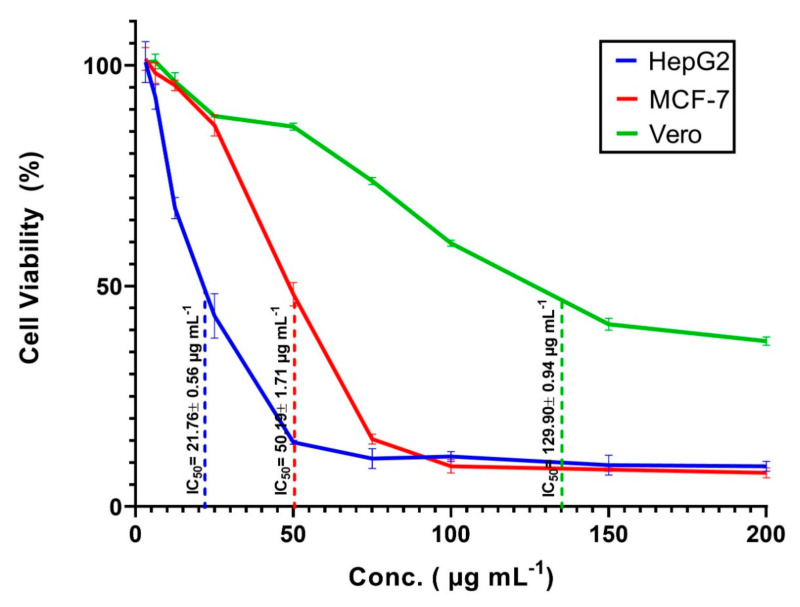
In vitro cytotoxic effects of Ag-NPs fabricated with the callus extract of *S. incanum* L. against two cancerous cells HepG2 and MCF-7. The data are statistically different at *p* ≤ 0.05, (*n* = 3); error bars are means ± SE (standard error).

The incorporation of nanomaterials especially Ag-NPs, in various materials of daily use such as cosmetic products, medical textiles, health care products, wound healing substances, and drug carriers was achieved. Additionally, Ag-NPs can be used as antitumor agents to prevent the proliferation of cancer cells [78]. As a result of this high usage rate, humans are exposed to these materials at different doses and times. Therefore, it is necessary to assess the toxicity of these materials on human or animal cell lines. The in vitro MTT assay method is a sensitive and accurate method used to assess cell viability and proliferation due to exposure to external substances. In this study, the cell viability of two types of cancerous cells, HepG2 and MCF-7 and one type of normal cell, Vero cell line, were exposed to different concentrations (200, 150, 100, 75, 50, 25, 12.5, 6.25, and 3.125 µg mL^−1^) of callus mediated synthesized Ag-NPs and assessed after 48 h. The microscopic investigation of treated cells showed that the cell shape was altered, a partial or complete loss of the monolayer structure, some granulations, and rounding or shrinking of the cells as compared with untreated cell lines (Figure 8). Our results showed that the viability of cancerous cells was decreased by increasing the concentrations of Ag-NPs. Compatible with our finding, Xia et al. [79] reported that the viability of cancerous cells LS174T (human adenocarcinoma colon cell), A549 (lung adenocarcinoma cell), MCF-7 (breast cancerous cell), and SMMC-7721 (hepatocarcinoma cell) was dose-dependent on Ag-NPs fabricated with the callus extract of *Taxus yunnanensis*. In the present study, the data analysis showed that the IC_50_ of Ag-NPs was 21.76 ± 0.56, 50.19 ± 1.71, and 129.9±0.94 µg mL^−1^ for HepG2, MCF-7, and Vero cell line, respectively (Figure 9). Consistent with our results, Xia et al. [79], who reported that the IC_50_ of Ag-NPs fabricated by callus extract of *Taxus yunnanensis* was 81.39 µg mL^−1^ for normal human liver cell (HL-7702) as compared with IC_50_ for cancerous cells, A549 (IC_50_ = 40.3 µg mL^−1^) and MCF-7 (IC_50_ = 42.2 µg mL^−1^). In the present study, the higher IC_50_ of Ag-NPs synthesized by callus extract to normal Vero cell, suggested our biosynthesized Ag-NPs were highly toxic to cancerous cells than normal cells. Vivek et al. [80] reported that the IC_50_ of Ag-NPs fabricated using plant extract of *Annona squamosa* was 50 µg mL^−1^ for the MCF-7 cell line. Moreover, the obtained results are compatible with those recorded by He et al. [81], who reported that the IC_50_ of the HepG2 cell line treated with Ag-NPs synthesized by aqueous fruit extract of Chinese herbal *Cornus officinalis* for 48 h was 21.46 μg mL^−1^. On the other hand, data recorded by Wang et al. [82] showed that the IC_50_ of Ag-NPs fabricated by extract of *Cornus officinalis* under UV-radiation was 69.72 mg mL^−1^ for the HepG2 cancerous cell line. There is variation between the IC_50_ values recorded by He et al. [81] and Wang et al. [82], although the Ag-NPs synthesized using the same aqueous plant extract could be attributed to the bioactive metabolites involved in the plant extract used for reducing and capping Ag-NPs, particles sizes, or biosynthesis environmental conditions [82].

The cytotoxic induction facilitated by Ag-NPs may be due to the formation of reactive oxygen species (ROS), oxidative stress, and release Ag^+^ ions [83]. The production of ROS increased because of mitochondrial damage, which was caused due to the decreased content of adenosine triphosphate (ATP) in the treated cell caused by Ag-NPs [77]. The apoptosis of the treated cells caused by Ag-NPs could be related to the release of Ag^+^ entering the cytoplasm and causes DNA damage and protein denaturation due to the enhancement of ROS production [84]. Moreover, Park et al. [85] reported that the apoptotic effect of Ag-NPs could be due to cytoplasmic membrane disruption, enhancement of ROS production, and an increase in the leakage of lactate dehydrogenase enzymes, which ultimately causes cancerous cell damage. In the current study, our callus extract mediated biosynthesized Ag-NPs exhibited a significant in vitro cytotoxic effect against cancerous cells with dose-dependent concentrations. The current investigation needs more analysis to investigate the probable mechanisms that induce apoptotic effects and assess these mechanisms based on the size and concentrations of Ag-NPs.

### 3.5. Comparison Study

The data represented in Table 3 show the comparative efficacy of Ag-NPs synthesized with different callus extracts and reported in published studies with those synthesized in the current study. The data exhibit the efficacy of callus aqueous extract from different plant species to fabricate Ag-NPs of various sizes. For example, Satyavani et al. [86] reported the efficacy of callus extract of *Citrullus colocynthis* L. to form spherical Ag-NPs with a size of 75 nm and showed its antibacterial activities against pathogenic biofilm bacteria with varied clear zones. On the other hand, Ag-NPs 12–25 nm in size synthesized with *Hyptis suaveolens* callus extract exhibit in vitro cytotoxic efficacy against two cancerous cell lines, breast epithelial adenocarcinoma cells (MDA-MB-231) and prostate cancerous cells (PC-3), using the MTT assay method [13]. In this study, callus aqueous extract of *S. incanum* L. showed high efficacy to fabricate spherical Ag-NPs with varied sizes ranging from 15 to 60 nm. The formed Ag-NPs exhibit efficacy against Gram-positive and Gram-negative bacteria, as well as *Candida albicans*. Additionally, they showed a high potential to inhibit the growth of some phytopathogenic fungi as well as in vitro cytotoxic efficacy against cancerous cell lines. Thus, callus extract mediated biosynthesis of NPs can be recommended as a safe, eco-friendly, and scalable approach to the synthesis active compounds with various biotechnological and biomedical applications.

## 4. Conclusions

Green chemistry has been the subject of growing interest due to its being environmentally safe, inexpensive, biocompatible, and avoiding producing toxic by-products. In this study, the in vitro leaf callus induction of *S. incanum* L. grown on MS medium supplemented with 1.0 mg^−1^ BA and 1.0 mg^−1^ 2,4-D, was used as a biocatalyst to reduce and stabilization of silver-to-silver nanoparticles. The color change from pale yellow to yellowish-brown indicated the formation of Ag-NPs, confirms via surface plasmon resonance detection at 440 nm. Moreover, the roles of metabolites present in callus extract in the bio-fabrication process were investigated using FT-IR analysis. The XRD and TEM analyses confirm the formation of crystalline and spherical Ag-NPs with sizes ranging from 15 to 60 nm. The callus mediated green synthesized Ag-NPs showed superior performance in antimicrobial activities against pathogenic Gram-positive and Gram-negative bacteria, and unicellular fungi. Additionally, they exhibited efficiency against plant pathogenic fungi with inhibition percentages of 76.3 ± 3.7, 88.9 ± 4.1, 67.8 ± 2.1, and 76.4 ± 1.0% for *Alternaria alternata*, *Fusarium oxysporum*, *Aspergillus niger*, and *Pythium ultimum*, respectively. Notably, green synthesized Ag-NPs have high efficacy in the treatment of two cancerous cell lines, HepG2 and MCF-7 at low concentrations (IC_50_ = 21.76 ± 0.56 and 50.19 ± 1.71 µg mL^−1^, respectively), while they effect on normal Vero cell line at high NPs concentrations (IC_50_ = 129.9 ± 0.94 µg mL^−1^). Based on obtained data, it can be concluded that the potentiality of callus extract in the biosynthesis of Ag-NPs has potential for different biomedical applications.

## Figures and Tables

**Table 1:** Effects of BA and 2.4-D in MS medium on callus induction frequency from leaves and petioles of *Solanum incanum* L. after five weeks of culture.

MS Supplemented with mg/L		Callus Induction Percentages (%)	
BA	2,4-D	Petioles	Leaves
0	0	0%	0%
0.5	1.0	80%	76.66%
0.5	1.5	60%	56.66%
0.5	2.0	83.33%	73.33%
1.0	1.0	86.66%	90%
1.0	1.5	46.66%	36.66%
1.0	2.0	33.33%	26.66%
1.5	1.0	53.33%	50%
1.5	1.5	26.66%	20%
1.5	2.0	16.66%	13.33%

**Table 2:** Effect of MS medium supplemented with different concentrations of BA and 2,4-D on fresh weight, dry weight (g/jar), and moisture content (%) of calli produced from leaves and petioles of *Solanum incanum* L. after five weeks of culture.

MS Supplementedwith mg L^−1^		Explants					
		Petioles			Leaves		
BA	2,4-D	Fresh Weight (g/jar)	Dry Weight (g/jar)	Moisture Content (%)	Fresh Weight (g/jar)	Dry Weight (g/jar)	Moisture Content (%)
0	0	0	0	0 %	0		0 %
0.5	1.0	4.08	0.30	92.64%	4.18	0.29	93.06%
0.5	1.5	2.63	0.23	91.25%	2.77	0.26	90.61%
0.5	2.0	3.11	0.29	90.67%	3.15	0.28	91.11%
1.0	1.0	4.68	0.32	93.16%	5.13	0.37	92.78%
1.0	1.5	1.86	0.11	94.08%	2.08	0.20	90.38%
1.0	2.0	1.33	0.10	92.48%	1.41	0.12	91.48%
1.5	1.0	2.16	0.19	91.20%	2.43	0.25	89.71%
1.5	1.5	1.08	0.09	91.66%	1.16	0.10	91.37%
1.5	2.0	0.9	0.08	91.11%	1.10	0.09	91.81%

**Table 3:** A comparison study between Ag-NPs synthesized by different callus extracts and those synthesized in the current study.

Callus Aqueous Extract Mediated Biosynthesis of Ag-NPs	Shape	Size	Applications	Reference
*Citrullus colocynthis* (L.)	Spherical	75 nm	Antibacterial activity	[86]
*Allophylus serratus*	Spherical	42 to 50 nm	Antibacterial activity	[56]
*Centella asiatica*	Spherical	5–40 nm	Antibacterial activity	[87]
*Hyptis suaveolens*	Spherical	12 to 25 nm	In vitro cytotoxicity against cancer cells	[13]
*Gymnema sylvestre*	Spherical	3–30 nm	Antifungal activity against Candida spp.	[18]
*Taxus yunnanensis*	Spherical	6.4 to 27.2 nm	Antibacterial activityIn vitro cytotoxicity	[79]
*Sesuvium portulacastrum* L.	Spherical	5 to 20 nm	Antibacterial and antifungal activities	[88]
*Cinnamomum camphora*	Spherical	5.47–9.48 nm	Antibacterial activity	[42]
*Solanum incanum* L.	Spherical	15–60 nm	Antibacterial and anticandidalAnti-phytopathogenic fungiIn vitro cytotoxicity	This study

## Data Availability

The data presented in this study are available on request from the corresponding author.

## Supplementary Materials

The following are available online at https://www.mdpi.com/2218-273X/11/3/341/s1. Figure S1: Antimicrobial activity of different concentrations of Ag-NPs synthesized by *Solanum incanum* L. against *Bacillus subtilis* (A); *Staphylococcus aureus* (B); *Pseudomonas aeruginosa* (C); *Escherichia coli* (D); *Candida albicans* (E); *Klebsiella pneumoniae* (F). Figure S2. Antifungal activity of different Ag-NPs concentration (200, 150, 100, 50, and 25 µg mL^−1^) against plant pathogenic fungi, *Alternaria alternata* (A); *Fusarium oxysporum* (B); *Aspergillus niger* (C); *Pythium ultimum* (D).
