# Supplementary material for: Antimicrobial and In Vitro Cytotoxic Efficacy of Biogenic Silver Nanoparticles (Ag-NPs) Fabricated by Callus Extract of Solanum incanum L
Source: Biomolecules. 2021 Feb 24;11(3):341. doi: 10.3390/biom11030341 (PMC7996206; doi:10.3390/biom11030341)
Supplement: Supplementary file 1 [file biomolecules-11-00341-s001.pdf]

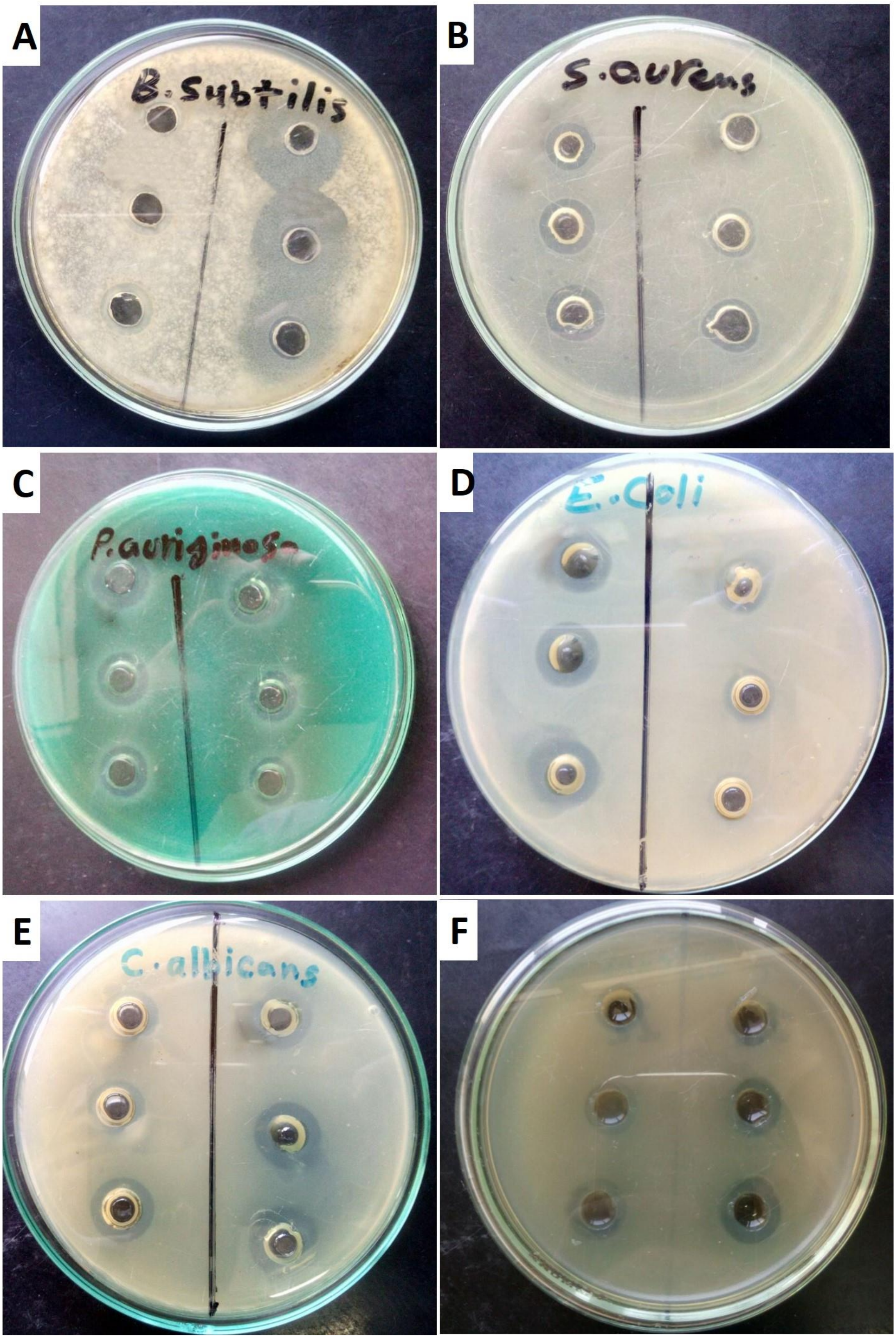

Figure S1. Antimicrobial activity of different concentrations of Ag-NPs synthesized by *Solanum incanum* L against *Bacillus subtilis* (A); *Staphylococcus aureus* (B); *Pseudomonas aeruginosa* (C); *Escherichia coli* (D); *Candida albicans* (E); and *Klebsiella pneumoniae* (F).

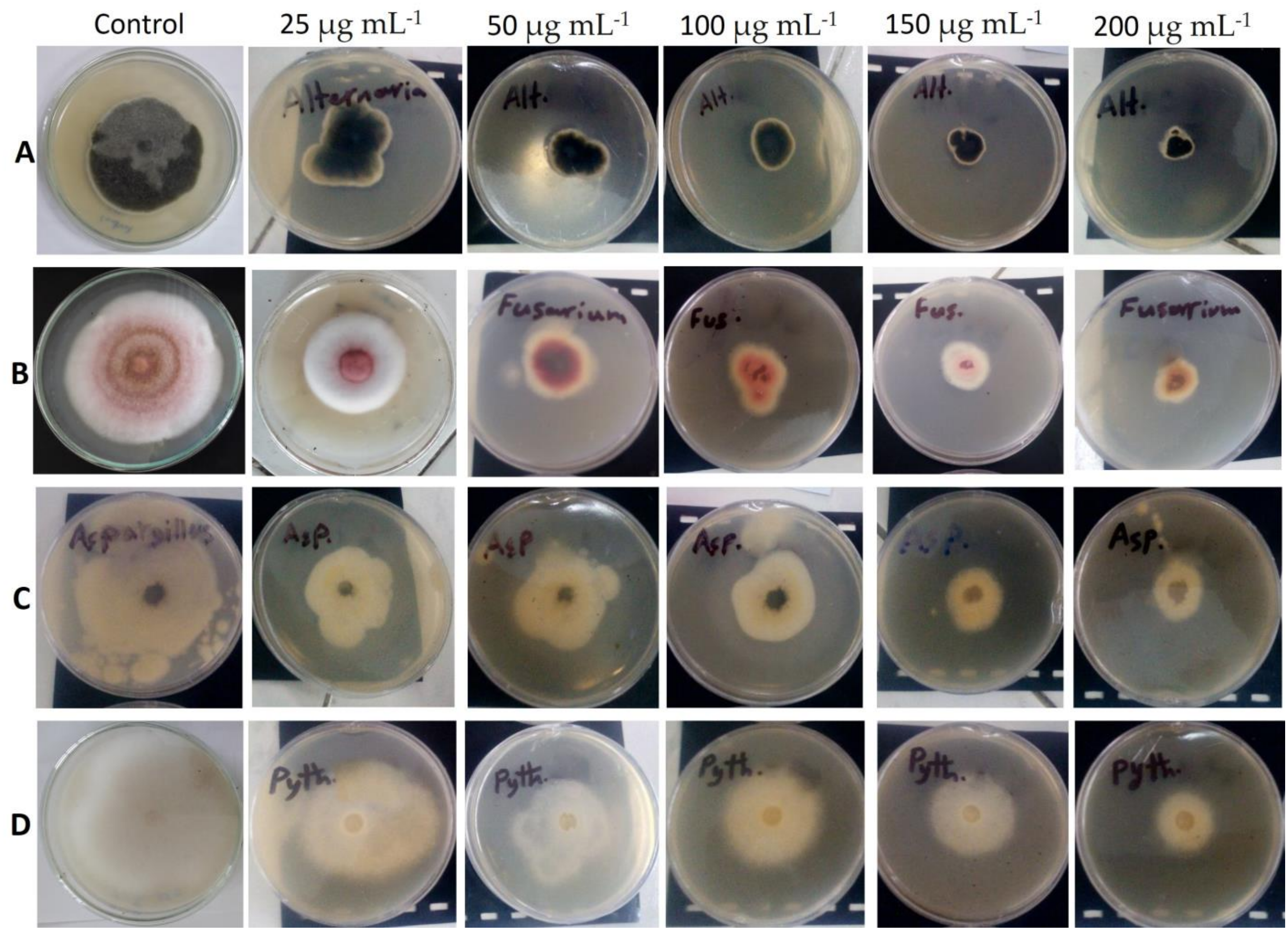

Figure S2. Antifungal activity of different Ag-NPs concentration (200, 150, 100, 50, and 25 μg mL^{-1}) against plant pathogenic fungi, *Alternaria alternata* (A); *Fusarium oxysporum* (B); *Aspergillus niger* (C); and *Pythium ultimum* (D). .
